# Effect of dietary inclusion of *Pennisetum purpureum* (Napier) grass on growth performance, rumen fermentation and meat quality of feedlot sussex red steers

**DOI:** 10.1007/s11250-024-03959-3

**Published:** 2024-04-20

**Authors:** T P Rabatseta, P Fourie, B D Nkosi, I M M Malebana

**Affiliations:** 1 Agricultural Research Council-Animal Production, Irene Campus, Private Bag X2 − Irene 0062, Pretoria, South Africa; 2 Central University of Technology, Free State Private Bag X20539, Bloemfontein, 9300 South Africa

**Keywords:** Fattening diet, Fibre source, Ruminants, Carcass characteristics, Beef

## Abstract

The aim of this study was to evaluate the growth performance, fermentation indices and meat quality of Sussex steers fed totally mixed rations that composed of graded inclusion levels of Napier grass (NP). Three experimental diets designated as diet 1 (0.0 g kg^–1^ NP: Control), diet 2 (300 g kg^–1^ NP grass) and diet 3 (600 g kg^–1^ NP) were formulated. Twenty-four male steers aged 8 months with an average body weight of 185.0 ± 30 kg were used. In a completely randomized design, the animals were allocated to the diets and fed for 120 days. Dietary NP inclusion reduced (*P* < 0.05) the animals’ average daily gain and increased the feed efficiency. The steers’ daily feed intake and final body weight decreased (*P* < 0.05) with a 600 g kg^–1^ inclusion level. The fermentation indices were not affected (*P* > 0.05) by the inclusion. While the inclusion reduced (*P* < 0.05) warm muscle temperature, it had no effect (*P* > 0.05) on carcass dressing percentage, warm and cold initial and ultimate pH. However, 600 g kg^–1^ inclusion level reduced (*P* > 0.05) warm and cold carcass weights. Meat physical attributes, moisture characteristics and tenderness were not affected (*P* > 0.05) by dietary treatments, except for the 7-days aged meat thaw loss, which increased at 600 g kg^–1^ inclusion level. Inclusion of 300 g kg^–1^ increased meat protein and fat, but dry and organic matter contents decreased with increasing inclusion levels. Dietary inclusion of NP grass up to 300 g kg^–1^ in steers’ diets improved feed intake, carcass traits and yielded meat high in protein and fat.

## Introduction

There are numerous different roughage sources available for use in ruminant feedlot fattening diets within the South African feedlot industry. These sources include *Eragrostis curvula* (weeping love grass), corn silage, wheat bran/straw, bagasse and cottonseed hulls (Vermaak, 2011). Roughage in fattening diets is utilized as a source of functional fibre that stimulates saliva production and chewing (Vermaak, 2011). The quantity of roughage within a ration elicit feed intake and maintain digestive health to maximize net energy for gain (NEg) in animals (Jardstedt et al., 2020).

The *Eragrostis curvula* grass (Weeping lovegrass), in the form of hay, is one of the commonly used roughage in South Africa (SA), with a mean fibre fraction of 84.45% neutral detergent fibre (aNDF), 50.27% acid detergent fibre (ADF) and 76.3% acid detergent lignin (ADL) (Gemede, 2014); as well as a mean crude protein content of less than 60 g kg^–1^, which may require supplementation with other protein sources such as urea (Tesfayohannes et al., 2013). Its preference is based on its ability to adapt to high temperature and radiation; tolerate drought and capacity to grow in sandy soils (Carballo et al., 2019). This grass is highly valued by farmers particularly for cattle feed in semi-arid regions (Habte et al., 2022). Despite its preference as a feed resource in SA, Weeping lovegrass is a C_4_ perennial grass that has threatened natural ecosystems globally (Roberts et al., 2021). Additionally, like other C_4_ species, Weeping lovegrass has lower nutritional quality compared to C_3_ species.

Another alternative source of roughage in SA is production of silage from corn. However, production of silage from corn is challenging due to the competition between animals and humans for corn (Wilkinson and Lee, 2018). Additionally, the use of the agricultural agro-industrial by-products such as wheat bran/straw, bagasse and cottonseed hulls as roughage sources is limited by their composition variability, severe seasonality, and low in production and often contain undesirable contaminants of organic and inorganic origin (Yang et al., 2021). Given the above challenges, it is imperative to search for alternative roughage ingredients that can be used as sources of fibre in ruminant fattening diets.

Napier grass (*Pennisetum purpureum*), is one of the highest yielding perennial grass that has been widely used as a tropical forage, producing greater biomass yields throughout the year compared to other cultivated or natural grasses (Sinche et al., 2021). Napier grass is presently the most potential roughage grass in animal production systems, with its remarkable characteristics; including regrowth ability, high yield and drought tolerance (Fukagawa and Ishii, 2018). When supplemented with legumes and other protein concentrates, Napier grass can provide a satisfactory source of fibre for ruminants (Islam et al., 2024).

According to Rahman et al. (2014), this grass contains (g kg^− 1^ DM) 240 dry matter, 95 crude protein, 557 neutral detergent fibre, and 7.5 MJ metabolizable energy. The quality and quantity of feedstuff (roughages) affect feed intake, body weight gain and meat taste of ruminants (Papa, 2021). In a study feeding diets composing of Napier, *Brachiaria* or *Leucaena leucocephala* as roughage to goats, results showed that feed intake was not affected (P0 > 0.05). Weight gains were high (*P* < 0.05) in goats fed Napier and *Brachiaria* based diets. However, the diets had no significant effect (*P* > 0.05) on the taste, juiciness or overall acceptability of the meat (Papa, 2021).

The use of Napier grass in steers fattening diet to replace Weeping lovegrass as a roughage, either completely or partially is not well researched. Additionally, the effects of different dietary inclusion levels of Napier grass on growth performance, fermentation dynamics and quality of meat from fattening steers are not known. More so, both low (300 g kg^− 1^) and high (600 g kg^− 1^) dietary inclusion levels of Napier grass as source of fibre in fattening diets is worth documenting. It is therefore imperative to determine the optimal dietary inclusion level at which Napier grass in fattening diet can improve growth performance and quality of meat from steers. Thus, the authors’ objective was to evaluate graded levels of Napier grass as a dietary source of fibre on growth performance, fermentation dynamics, carcass characteristics and meat quality of steers.

## Materials and methods

### Study location

The experiment (Ethic clearance APEC 21/13) was carried out at the Agricultural Research Council – Animal Production in Irene, Pretoria, South Africa (longitude 25º 53’ 59.6” S, latitude 28º 12’ 51.6” E).

### Chemical analysis of the forages and dietary treatments

The proximate [dry matter (DM), organic matter (OM), crude protein (CP), ether extract (EE) and gross energy (GE)] and fibre fraction [neutral detergent fibre (aNDF), acid detergent fibre (ADF), and acid detergent lignin (ADL)] as well as calcium and phosphorus analysis of the *Pennisetum purpureum, Eragrostis curvula* grasses, and that of the dietary treatments were done following standard methods. The proximate assay was carried out according to AOAC (2006) using methods 930.15, 942.05, 984.01, 920. 39, respectively. For gross energy, an MC-1000 Modular Calorimeter (Energy Instrumentation, Centurion, South Africa) equipped with a PC and MC1000 software was used. The fibre fractions were determined according to methods of Van Soest et al. (1991).

### Feed ingredients and diet formulation

The *Pennisetum purpureum* (Napier grass) was planted in April 2021 at SMR farm situated near the Vaal Dam, in South Africa. The coordinates of the farm are 26°53’40.99” S 28°08’43.98” E. Minimum temperatures are 4.3 ºC in winter and 16.2 ºC in summer, while maximum temperatures are 16.7 ºC in winter and 25.6 ºC in summer. The average rainfall is 4 mm in winter and 132 mm in summer. The grass was harvested when it reached between 1.5 and 2 m height at 6 to 8 weeks in August and September 2021 using a Feraboli 945 forage harvester (Fondata Nel, Cremona, Italy) adjusted to achieve a 10 mm theoretical chop length. Following harvest, the grass was dried on the sun for 3 days. Representative samples of Napier grass were collected and analysed for chemical composition (proximate, fibre fraction and mineral composition).

*Eragrostis curvula* hay was sourced from the Agricultural Research Council in Roodeplaat, Gauteng Province. Ingredients such as wheat bran, soyabean meal, hominy chop, salt, urea, feedlime and premix were sourced Obaro in Tshwane, Gauteng Province. Using the ingredients, experimental diets were formulated as per guidelines of the National Research Council (NRC, 1996) requirements for fattening cattle. The experimental diets were formulated as: Diet 1 consisted of 0 g kg^− 1^ DM Napier grass, while Diets 2 and 3 consisted of 300 and 600 g kg^− 1^ DM Napier grass, respectively, (Table 1).

**Table 1:** Feed ingredients and formulation of the treatment diets on DM basis

Ingredients (%)	Diet 1	Diet 2	Diet 3
*Pennisetum purpureum*	0	30.2	60
Maize meal/hominy chop	56.0	42.8	26
Wheaten bran	14.8	14.6	-
Molasses meal	9.9	9.6	11.5
*Eragrostis curvula* hay	9.9	-	-
Soybean oil cake meal	4.9	-	-
Feed lime	1.5	1.0	1.24
Salt (grade 1 cattle salt)	0.5	0.5	0.5
Feed grade Urea	1.5	0.2	0.2
Vit.Min premix	1.5	0.2	0.2

Vit.min premix: 6500 IU vitamin A, 1200 IU vitamin D3, 40 IU vitamin E, 2 mg vitamin K3, 1–5 mg vitamin B1, 4.5 mg vitamin B2, 0.03 mg vitamin B12, 2.5 mg vitamin B6, 25 mg niacin, 12 mg calcium pantothenate, 190.5 mg choline, 0.6 mg folic acid, 0.05 mg biotin, 40 mg manganese, 100 mg zinc, 125 mg copper, 1 mg iodine, 100 mg ferrous and 0.3 mg selenium. Diet 1, control (0 g kg−1); diet 2, 300 g kg−1 Napier grass; diet 3, 600 g kg−1 Napier grass

**Table 2:** Chemical composition of *Pennisetum purpureum* and *Eragrostis curvula* (*n* = 3)

Parameter	*Pennisetum purpureum*	*Eragrostis curvula*
**Proximate (%)**		
DM	93.18	93.7
OM	93.30	93.01
CP	12.00	4.5
Ether Extract	2.22	1.86
Gross energy (MJ/kg DM)	16.39	18.62
**Fibre fraction (%)**		
aNDF	62.65	78.9
ADF	36.38	43.2
ADL	4.37	7.0

DM= dry matter, OM = organic matter, CP = crude protein, NDF = neutral detergent fibre, ADF = acid detergent fibre, ADL = acid detergent lignin

### Animal management and study design

The Sussex red steers were attained from a farmer in the Free State Province, South Africa and were ear-tagged and individually weighed before quarantined for 14 days. Twenty-four (24) male steers of 8 months old with an average initial body weight of 185.0 ± 30 kg (mean ± SD) were used for this study. Steers were adapted to the environment and experimental diets for 14 days prior to collection of the study data. The steers were individually allocated to 3 experimental (treatment) diets with 8 steers per treatment in a completely randomized design. The steers were then fed the 3 treatment diets for 120 days during which data was collected on daily basis.

### Data collection, slaughtering and sample collection

The steers were weighed in the beginning and weekly to determine body weight gain. The difference between the quantities of feed offered daily to the steers and leftovers was calculated to determine daily feed intake. Feed conversion ratio was calculated by dividing feed intake over weight gain. Following the growth study, the steers were fasted for 24 h before being humanely slaughtered according to South African standards (Hoffman et al., 2003). After slaughter, the steers were eviscerated during which rumen digesta samples were collected for the determination of pH. The samples were then sieved to harvest rumen liquor for volatile fatty acids (VFAs) determination. Characteristics of the carcasses were evaluated followed by chilling of each carcass overnight. Subcutaneous fat thickness was measured using a Vernier caliper (150 mm Krekeler electronic digital caliper, Helmut Zepf Medizin Technik GmbH, Seitingen Oberflacht, Germany). Meat samples were cut from *Longissimus dorsi* muscle for the determination of physical attributes, moisture characteristics, and tenderness and proximate.

### Statistical analyses

Data on the chemical composition of the forages and treatment diets; growth performance, rumen fermentation dynamics, carcass and meat quality were subjected to ANOVA and analysed using Genstat statistical software (Genstat, 2000). The Fisher’s least significant difference (LSD) test was used to separate mean differences. The probability value was tested at 5%. Data were fitted in the following model:

$${{\text{Y}}_{{\text{ij}}}}\,=\,{\text{\varvec{\upmu}}}\,+\,{{\text{T}}_{\text{i}}}\,+\,{{\text{\varvec{\upvarepsilon}}}_{{\text{ij}}}}$$

Where Y_ij_ is the individual observation of the i^th^ dietary treatment and the j^th^ replicate, µ is the overall mean, T_i_ is the fixed effect of the i^th^ dietary treatment (i = 1,2,3), ε_ij_ is the random residual error.

## Results

Table 1 illustrate the feed ingredients and the quantities used to formulate the treatment diets. *Pennisetum purpureum* (Napier grass) replaced *Eragrostis curvula* (Weeping lovegrass) in a totally mixed ration as a source of fibre at different inclusion levels. The chemical composition of Napier grass and Weeping lovegrass is shown in Table 2. While the crude protein content was higher (*P* < 0.05) in Napier grass than in Weeping lovegrass, gross energy and fibre fraction content were higher (*P* < 0.05) in Weeping lovegrass relative to Napier grass. Chemical composition of the treatment diets is demonstrated in Table 3. The GE content was high (*P* < 0.05) in the diets consisting of 0 and 300 g kg^− 1^ Napier grass than that consisting of 600 g kg^− 1^ Napier grass. While the control diet had high (*P* < 0.05) DM content, 300 g kg^− 1^ Napier grass based diet had high (*P* < 0.05) fat content and 600 g kg^− 1^ Napier grass based diet had higher (*P* < 0.05) ash content. The CP content was significantly similar (*P* > 0.05) across the treatment diets. Increasing Napier grass inclusion level reduced (P˂0.05) the daily feed intake, which subsequently reduced daily gains and final body weight of the steers (Table 4). In addition, steers fed the diet that contained 600 g kg^− 1^ Napier grass had the highest (*P* < 0.05) feed efficiency compared to the steers in other diets. Results for rumen fermentation of steers fed different inclusion levels of Napier grass are shown on Table 5. The rumen digesta pH of the steers were similar (*P* > 0.05) across the treatment diets. There was no significant difference (*P* > 0.05) in the rumen fluid volatile fatty acid content of the steers among the dietary treatments. Carcass traits of the steers are on Table 6. The results show that increasing dietary inclusion levels of Napier grass reduced (P˂0.05) the warm and cold carcass weight, and warm and cold muscle temperature of the steers. However, the dressing percentages of the carcasses as well as the warm and cold muscle pH were not affected (*P* > 0.05) by the dietary treatments. Dietary inclusion of Napier grass in fattening diets did not affect (*P* > 0.05) the drip loss, colour and myoglobin redox reaction of the meat from steers (Table 7). Thaw losses for meat aged for 7 days increased with increasing dietary inclusion level of Napier grass in steers fattening diets, while cooking loss, water holding capacity and warner Bratzler shear force of meat from steers were similar (*P* > 0.05) across dietary treatments (Table 8). The results for the sarcomere length (SL) and myofibrillar fragmentation length (MFL) are illustrated in Table 9, and were not affected (*P* > 0.05) by the dietary treatments. Increasing the inclusion level of Napier grass in steers fattening diets to 600 g kg^− 1^ reduced (*P* < 0.05) the DM and OM of the meat. The meat CP and fat content in the 300 g kg^− 1^ diet was comparable (*P* < 0.05) to that in the control diet (0 g kg^− 1^) Napier included diet (Table 10).

**Table 3:** Chemical composition of the treatment diets (*n* = 3)

Parameter	Diet			LSD	SE	P
	Diet 1	Diet 2	Diet 3			
**Proximate (%)**						
Dry matter	88.61^a^	84.29^c^	86.85^b^	0.288	0.144	˂0.001
Crude protein	11.24^ab^	11.75^a^	10.93^b^	0.612	0.307	0.045
Ash	7.49^b^	4.15^c^	13.74^a^	0.673	0.337	˂0.001
Fat	2.19^b^	2.52^a^	1.31^c^	0.11	0.06	˂0.001
Gross energy (MJ g/kg)	14.99^a^	15.26^a^	13.82^b^	0.622	0.203	0.010
**Fibre fraction (%)**						
Neutral detergent fibre	46.44^a^	30.30^b^	46.37^a^	4.654	2.330	˂0.001
Acid detergent fibre	26.82^a^	15.28^c^	23.19^b^	0.810	0.406	˂0.001
Acid detergent lignin	15.67^a^	8.05^c^	11.15^b^	1.040	0.521	˂0.001
**Macro-minerals (100 g/kg)**						
Calcium	11.37^c^	17.78^a^	13.82^b^	0.552	0.174	˂0.001
Phosphorus	7.59^b^	11.64^a^	10.89^a^	1.056	0.332	0.002

a, b,c within rows means with different superscripts differ significantly (P˂0.05). DM – dry matter. Diet 1, control (no Napier grass); diet 2, 300 g kg−1 Napier grass; diet 3, 600 g kg−1 Napier grass LSD – least significant difference, SE – standard error, P – probability value

**Table 4:** Effect of dietary inclusion level of *Pennisetum purpureum* on growth performance of Sussex red steers (*n* = 8)

Parameters		Diet		LSD	SE	P
	Diet 1	Diet 2	Diet 3			
Initial body weight (kg)	182.4	184.2	188.3	7.73	6.89	0.290
Final body weight (kg)	399.6^a^	379.6^a^	294.16^b^	22.47	20.01	˂0.001
ADG (g/day)	1.80^a^	1.72^b^	0.53^c^	1.18	0.16	˂0.001
DFI (kg/day)	6.78^a^	6.33^a^	4.47^b^	0.670	0.596	˂0.001
Feed efficiency (g/g)	3.78^c^	5.21^b^	8.69^a^	0.996	0.887	˂0.001

^a, b,c^ within rows means with different superscripts differ significantly (P˂0.05). Diet 1, control (0 g kg−1); diet 2, 300 g kg−1 Napier grass; diet 3, 600 g kg−1 Napier grass. ADG – average daily gain, DFI –daily feed intake, LSD – least significant difference, SE – standard error, P – probability value

**Table 5:** Effect of graded dietary inclusion level of *Pennisetum purpureum* on rumen fermentation dynamics of Sussex red steers (*n* = 8)

Parameter		Diet		LSD	SE	P
	Diet 1	Diet 2	Diet 3			
Rumen digesta pH	6.7	6.6	6.6	0.31	0.28	0.980
**Volatile fatty acid (g/L)**						
Acetic acid	42.4	48.0	40.1	17.32	15.36	0.616
Butyric acid	2.98	4.06	3.50	1.288	1.142	0.237
Propionic acid	8.49	13.20	8.41	7.71	6.83	0.347
Valeric acid	0.50	0.79	0.48	0.44	0.39	0.283
Isobutyric acid	1.30^b^	1.73^a^	1.51^ab^	0.38	0.34	0.088
Isovaleric acid	2.05	2.75	2.38	0.830	0.736	0.239

a, b within row means with same superscript did not differ significantly (P˃0.05). LSD – least significant difference, SE – standard error, P – probability value. Diet 1, control (0 g kg−1); diet 2, 300 g kg−1 Napier grass; diet 3, 600 g kg−1 Napier grass

**Table 6:** Effect of graded inclusion level of *Pennisetum purpureum* on carcass characteristics, pH and temperature of muscles from Sussex red steers (*n* = 8)

Parameter		Diet		LSD	SE	P
	Diet 1	Diet 2	Diet 3			
Warm carcass weight (kg)	218.0^a^	204.4^a^	160.7^b^	15.02	13.37	˂0.001
Cold carcass weight (kg)	213.4^a^	199.7a^a^	158.2^b^	14.46	12.88	˂0.001
Dressing percentage (%)	54.70	53.81	54.55	2.260	2.013	0.682
Warm muscle pH_i_	6.00^ab^	6.04^a^	5.95^b^	0.08	0.07	0.077
Cold muscle pH_u_	5.58^a^	5.60^ab^	5.51^b^	0.08	0.07	0.081
Warm muscle temperature (°C)	39.46^a^	38.19^b^	37.69^b^	0.974	0.868	0.004
Cold muscle temperature (°C)	11.51^a^	12.60^a^	5.34^b^	3.653	3.253	˂0.001

a, b Within row means with the same superscripts are significantly different (P ˂0.05). LSD – least significant difference, SE – standard error, P – probability value. Diet 1, control (0 g kg−1); diet 2, 300 g kg−1 Napier grass; diet 3, 600 g kg−1 Napier grass

**Table 7:** Effect of graded inclusion level of *Pennisetum purpureum* on drip loss, colour and myoglobin redox reaction from Sussex red steers (*n* = 8)

Diet				LSD	SE	P
Parameter	Diet 1	Diet 2	Diet 3			
Drip loss (%)	1.84	2.04	1.83	1.064	0.948	0.899
L*(D_65_)	32.24	30.49	34.74	5.018	4.468	0.230
a* (D_65_)	9.75	8.11	8.35	2.066	1.839	0.224
b* (D_65_)	11.44	9.57	10.39	2.590	2.306	0.334
C* (D_65_)	15.05	12.57	13.38	3.259	2.902	0.289
H* (D_65_)	49.16	49.50	51.08	2.901	2.583	0.361
**Myoglobin redox reaction (%)**						
Metmyoglobin	2.71	2.71	2.57	2.316	2.063	0.989
Deoxymyoglobin	60.3	71.3	70.6	13.66	12.16	0.195
Oxymyoglobin	37.1	25.4	27.0	13.90	12.38	0.186

LSD – least significant difference, SE – standard error, P – probability value. Diet 1, control (0 g kg−1); diet 2, 300 g kg−1 Napier grass; diet 3, 600 g kg−1 Napier grass

**Table 8:** Water-holding capacity, WBSF, Cooking and thaw losses of meat from Sussex red steers fed dietary inclusion levels of *Pennisetum purpureum* (*n* = 8)

Parameter	Diet			LSD	SE	P
	Diet 1	Diet 2	Diet 3			
Water-holding capacity	0.31	0.37	0.32	0.07	0.06	0.208
**1-day aged meat**						
Thaw loss (%)	3.41^ab^	1.83^b^	4.91^a^	2.755	2.453	0.090
Cooking loss (%)	19.58	19.74	22.31	5.075	4.519	0.464
WBSF (kg)	3.94	3.61	4.19	0.989	0.881	0.477
**7 day aged meat**						
Thaw loss (%)	2.6^b^	1.31^b^	5.76^a^	1.930	1.719	< 0.001
Cooking loss (%)	21.65	18.45	20.98	4.095	3.646	0.249
WBSF (kg)	3.28	3.02	3.32	0.811	0.722	0.697

a, b Within row means with different superscripts are significantly different (P ˂ 0.05). LSD – least significant difference, SE – standard error, P – probability value. Diet 1, control (0 g kg−1); diet 2, 300 g kg−1 Napier grass; diet 3, 600 g kg−1 Napier grass

**Table 9:** Effect of graded inclusion level of *Pennisetum purpureum* on sarcomere length and myofibrillar fragmentation lengths from carcasses of Sussex red steers (*n* = 8)

Parameter (µm)	Diet			LSD	SE	P
	Diet 1	Diet 2	Diet 3			
Sarcomere length	1.93	2.02	2.00	0.09	0.08	0.135
**Myofibrillar fragmentation length**						
MFL 1 dpm	33.7	31.6	39.6	8.67	7.72	0.162
MFL 7 dpm	25.83	23.86	27.34	4.616	4.110	0.307

MFL − myofibrillar fragmentation length, dpm − days post−mortem, LSD – least significant difference, SE – standard error, P – probability value. Diet 1, control (0 g kg−1); diet 2, 300 g kg−1 Napier grass; diet 3, 600 g kg−1 Napier grass

**Table 10:** Effect of graded dietary inclusion level of *Pennisetum purpureum* on the proximate of beef from Sussex red steers (*n* = 8)

Parameters (%)	Diet			LSD	SE	P
	Diet 1	Diet 2	Diet 3			
Dry matter	32.86^a^	31.05^b^	27.78^c^	1.505	2.437	˂0.001
Organic Matter	31.84^a^	30.05^b^	26.81^c^	1.549	2.509	˂0.001
Protein	22.92^a^	22.18^a^	20.47^b^	1.383	2.241	0.003
Fat	8.97^a^	7.46^a^	5.51^b^	1.701	2.756	˂0.001

^a, b, c^ Within row means with different superscripts differ significantly at (*P*<0.05). LSD – least significant difference, SE – Standard Error, P – probability value. Diet 1, control (0 g kg−1); diet 2, 300 g kg−1 Napier grass; diet 3, 600 g kg−1 Napier grass

## Discussion

### Chemical composition

The nutrient compositions of Napier grass and that of Weeping lovegrass are presented in Table 2. Napier grass has higher content of crude protein and lipid relative to Weeping love grass, with the latter having higher (*P* < 0.05) gross energy and fibre fractions than the Napier grass. The higher content of protein in Napier than in Weeping love grass translate to Napier grass having the potential to also be used as a source of protein; while the higher energy and fractions of fibre in Weeping lovegrass points to its known source of energy and fibre in feed. The lower fibre content in Napier relative to that in Weeping love grass does not omit its potential to partially replace Weeping lovegrass in diets as a source of fibre. Studies have reported that high lignin content leads to reduced forage voluntary intake in cattle (Cavallini et al., 2023; Guimarães et al., 2023). Thus, the low ADL content in Napier grass (Table 2) show that the use of Napier grass in diets will not result in reduced voluntary feed intake and digestibility by ruminants.

Dietary inclusion of Napier grass at 300 g kg^− 1^ in fattening diets led to a diet with the lowest (*P* < 0.05) DM content compared to the control (0 g kg^− 1^ Napier grass) and the 600 g kg^− 1^ Napier based diet. The CP content of the treatment diets did not differ (*P* > 0.05). Both the treatment diets consisting of 0 and 300 g kg^− 1^ of Napier grass had higher GE content, while 600 g kg^− 1^ Napier grass based diet had higher (*P* < 0.05) ash content and 300 g kg^− 1^ Napier grass based diet had higher (*P* < 0.05) fat content than their counterparts. The nutritional value of forage influences forage utilization by animals, which subsequently affects animal production (Mirzaei-Aghsaghali and Maheri-Sis, 2011). Unlike mature animals, growing cattle require increased dietary protein in terms of quantity and quality to meet their high demand for amino acids required for muscle accretion (Lee et al., 2020). The GE concentration in feeds is mostly dependent on ash, CP and fat concentrations. The GE concentration will increase as ash concentration decreases and as CP or lipid concentrations increase (Cabezas-Garcia et al., 2021). Indeed results in this study show that the CP content in the treatment diets increased with decreasing ash concentration and increasing lipid and CP concentrations. This is an indication that the high CP, fat and GE contents in the diet that contained 300 g kg^− 1^ of Napier grass will enhance diet utilization to meet the amino acids required by the steers for growth.

Forages are necessary diet components for ruminants, as they provide the fibre needed to optimize microbial activity and rumen function (Turano et al., 2016). The fibre fractions of the Napier grass-based diets was lower (*P* < 0.05) compared to the control diet, which contained Weeping lovegrass (Table 3). The aNDF of the control diet was comparable to that of diet consisting of 600 g kg^− 1^ of Napier grass. The NDF plays a critical part in the ruminant digestive process, as it influences feed intake, rumen fermentation, and nutrient utilization. The ADF and ADL content were lowest (*P* < 0.05) in the 300 g kg^− 1^ Napier grass containing diet than that containing 600 g kg^− 1^of Napier grass. The high ADL content in the control diet can limit the digestibility of the diet due to the indigestible component of the roughage, whereas the lowest ADF and ADL content in 300 g kg^− 1^ Napier containing diet favours digestibility and enhance microbial activity, thus microbiome and rumen health of the steers. Improved microbial protein will lead to improved growth for the steers fed the 300 g kg^− 1^ Napier based diet.

Calcium (Ca) and phosphorus (P) are vital micro minerals for animal physiological functions that include protein synthesis, metabolic reactions, muscle contraction, impulses, enzyme activation, and maintenance of osmotic and acid-base balances, transmission of nerve as well as components in membranes (Ewing and Charlton, 2007). Results in this study show that the 300 g kg^− 1^ Napier grass based diet had a higher (*P* < 0.05) concentration of calcium and phosphorus compared to other diets. The high dietary concentration of Ca and P could enhance the physiological function of the animals fed diet containing 300 g kg^− 1^ of Napier grass. Improvement in physiological functions will not only improve the health of the animals, but also growth, which translate to more muscle accretion. Deficiencies in Ca and P in diets will otherwise result in metabolic disorders and poor growth.

### Growth performance

Feed intake is essential to ruminants for the maintenance of production. Results in this study show that feed intake (FI) and final body weight (FBW) of steers fed the control diet and the 300 g kg^− 1^ Napier grass containing diet were higher (*P* < 0.05) than those of steers fed diet containing 600 g kg^− 1^ of Napier grass. The better FI and FBW of the steers is attributed to the nutrient density of the two diets that were able to meet the nutrient requirements of the steers. Increasing dietary inclusion of Napier grass to 600 g kg^− 1^ reduced (*P* < 0.05) the average daily gain (ADG), while increasing the feed efficiency of the steers. The low ADG is attributed to the low GE content in the diet that was required for metabolic functions in steers. Moreover, reduced ADG in steers fed the 600 g kg^− 1^ Napier grass based diet is attributed to the high content of aNDF in the diet, which causes rumen filling that often leads to reduced feed intake. Hence, the final body weight of the steers fed the same diet was also low. The higher feed efficiency in steers fed the 600 g kg^− 1^ Napier grass based diet than other diets is as a result of the high quantity of nutrients that were required to meet the steers demand in order to be utilized for improved growth. The ADG of steers in this study followed an opposite trend to the feed efficiency. Hence, the efficiency at which the nutrients are being utilized by the steers is reduced while the FI and body weight gain are increased. Our results indicate that the growth of steers fed diets with Napier grass at 300 g kg^− 1^ was comparable to that of 0 g kg^− 1^ inclusion of Napier grass. Both treatment diets had high CP and GE content, which highlights the potential of Napier grass to be used in place of Weeping lovegrass in steers fattening diets on fibre basis owing to the nutrients adequacy of the dietary treatments. The nutrient-rich Napier grass could limit the use of other feed ingredients to meet the requirements of the ruminants and in return minimise feed and production cost.

### Fermentation indices

Table 5 shows the rumen fermentation indices of steers fed graded inclusion levels of Napier grass in place of Weeping lovegrass. Feeding ruminants on diets brings about rapid variations in the rumen microbiome. These variations include rumen liquor pH, which can range between 5.5 and 7.5 depending on the type of diet (Franzolin et al., 2010). The volatile fatty acids and rumen liquor pH results in this study were all similar across the treatments (*P* > 0.05). Although the concentration of Isobutyric acid in rumen digesta of steers fed the 300 g kg^− 1^ Napier included diet was high, it shared similarities with that from those fed diet with 600 g kg^− 1^, which also did not differ from the control diet. The rumen pH ranged between 6.6 and 6.7 (within the normal pH range for ruminants on concentrate and forage based diets). These similarities between the treatments attributes to steers having met their nutrient requirements and not in excess. Otherwise, one treatment would have showed a higher concentration than others would. Furthermore, these results indicate that inclusion of Napier grass in fattening diet did not perturb the fermentation indices of the diets, thus having the potential as a source of fibre in ruminants’ diets.

### Carcass traits

The weights of warm and cold carcasses from steers in the present study differed (*P* < 0.05) significantly amongst the diets (Table 6). Steers fed diet that contained 600 g kg^− 1^ Napier grass had lower (*P* < 0.05) warm and cold carcass weights compared to those fed the other diets. This was expected as the final body weight of steers fed the 600 g kg^− 1^ Napier grass diet followed the same trend as with the carcasses. Similar to the carcass weights, the cold muscle temperature of the steers fed the 600 g kg^− 1^ Napier grass diet was lower (*P* < 0.05) compared to its counterparts. Although the dressing percentage (DP), warm and cold muscle pHs of the carcasses were similar (*P* > 0.05) across the treatments, the warm muscle temperature decreased (*P* < 0.05) with increasing Napier grass inclusion. The average DP (54.3%) in this study is similar to that reported in Boran cattle by Mummed & Webb (2019). Consistently, Hattakum et al. (2019) obtained similar results when feeding steers on Napier grass mixed with pineapple silage.

The muscle pH in living animals is normally around 7.0 and a *post-mortem* pH decline occurs when the muscle pH reaches 5.4 due to accumulation of lactic acid produced during anaerobic glycolysis from glycogen (Chilanga, 2020). According to Kumar et al. (2022), ultimate pH higher than 5.6 indicates the susceptibility of the meat to microbial attack, which negatively affects the meat`s shelf life. The initial and ultimate pH of the carcasses from steers in the present study were similar (*P* > 0.05) and ranged between 5.1 and 5.6 indicating that inclusion of Napier grass in steers fattening diets at 300 and 600 g kg^− 1^ will not lower the shelf life of the meat from the fed steers. The pH values from carcasses in the present study did not exceed pH of 5.8, which is deemed dark, firm and dry (DFD) by Pophiwa et al. (2017) but closer to the value (5.8) recommended by Barrasso et al. (2022) for good quality meat.

Temperature in muscle is associated with increased myosin and sarcoplasmic protein denaturation, both of which contribute to shrinkage of myofilament lattice spacing (Hughes et al., 2018). A decline in temperature coupled with variation in ultimate pH of muscles *post-mortem* could account for the changes in meats’ technological and sensory qualities (Zhang et al., 2018). Good quality meat that is tender and juicy is characterized by a normal decline in temperature and pH. Our study shows that the inclusion of Napier grass at 0 and 300 g kg^− 1^ in fattening diets yielded better carcass traits compared to those from steers fed the 600 g kg^− 1^ inclusion level of Napier grass (Table 6).

### Meat physical attribute

Losses of various meat physical attributes such as drip, thawing, evaporation and cooking loss are among factors that influence the quality of meat (Devi et al., 2019). Drip loss, defined as the manifestation of liquid leakage from myofibres might result in loss of water, iron and proteins during the transition of muscle to meat (Kralik et al., 2018). Drip loss depends on the shortening of sarcomeres, which is influenced by the interaction of the muscle temperature, rigor development, and chilling condition (Devi et al., 2019). According to Traore et al. (2012), a drip loss that ranges from 2 to 4% is considered to be normal, while values below or above could be a sign of meat deterioration. Results of the present study show that meat drip loss was similar (*P* > 0.05) amongst dietary treatments (Table 7). The similarities in drip loss attest to the fact that the inclusion of Napier grass in the diets did alter the shortening of the sarcomere that would have otherwise caused a leakage on the meat. Moreover, the average drip loss of meat in this study was 2%, which is in agreement with the normal drip loss for beef reported by Traore et al. (2012).

Meat colour determines the consumers’ insight of product quality and thereby significantly affects the purchase decision (Ruedt et al., 2023). This is thus an important aspect for the meat industry. Meat appearance is based on a complex interplay between myoglobin and its chemical forms as well as light scattering from the muscle’s microstructure (Ruedt et al., 2023). Myoglobin is essentially responsible for the determination of meat colour (Suman and Joseph, 2014). Meat colour attributes (L*, a*, b*, chroma, hue angle) as well as the myoglobin state (met-, de- and oxymyoglobin) of meat in this study were similar (*P* > 0.05) across dietary treatments (Table 7). Since rapid decline in muscle pH and temperature have an effect on the meat colour, the similarities in meat colour traits and myoglobin redox reaction confirms that dietary inclusion of Napier grass did not alter the chemical forms of myoglobin, just as the muscle pH and temperature were within the recommended values for good quality meat. The normal meat physical attributes translate to prolonged meat shelf life that is acceptable to consumers based on the physical attributes. Undesirable attributes can shorten the shelf of the product, thus rejection by consumers.

### Moisture characteristic, water holding capacity and warner bratzler shear force

The moisture characteristics, water holding capacity and Warner Bratzler shear force of the meat from steers fed diets with graded levels of Napier grass is shown in Table 8. Meat moisture can be determined in numerous ways, including cooking loss, thaw loss, WHC and total moisture content. Loss of meat moisture is unavoidable *post-mortem* due to a decline in pH (Dang et al., 2021). Cooking loss refers to the reduction in weight of meat during the cooking process (Pang et al., 2020). The main components of cooking loss include thaw, drip and evaporation (Strydom et al., 2016). Thaw loss implies to the loss of meat fluid subsequent to the formation of exudates after freezing and thawing (Arshad et al., 2023). Results in the current study show that thaw loss of meat aged for 1 day and cooking losses of meat aged for 1 and 7 days did not differ (*P* > 0.05) across the diets. However, thaw loss for 7-days aged meat from steers fed diets with the highest inclusion level (600 g kg^− 1^) of Napier grass was higher (*P* < 0.05) than that from meat of steers fed the other diets. According to Leygonie et al. (2012), thawing is known to increase the amount of water lost in meat, most likely because of changes to the structure of the muscle fibres and/or denaturation of proteins. Our results suggest that inclusion of Napier grass at 600 g/kg^− 1^ in fattening diet resulted in high thaw loss due to denaturing of proteins, which brings about variation in the structure of the muscle fibres.

Water holding capacity (WHC) is the ability of meat to rapidly hold to its own or added water during processing, and it is an important component as it provides desirable traits to meat (Warner, 2014). In the present study, the meat WHC did not differ (*P* > 0.05) amongst the diets. According to Szmańko et al. (2021), WHC is measured by drip loss. By virtue of the dietary Napier grass inclusion having not impaired the drip loss of the meat, WHC was not different, implying that the inclusion of Napier grass in diets did not compromise the capacity of the meat to hold water.

A general agreement exist in literature that meat tenderness increases with freezing and thawing when measured with peak force (Szmańko et al. (2021). An increase in meat tenderness is correlated to the extent of frozen storage and the degree to which the meat is aged prior to freezing. Grayson et al. (2014) reported that the tenderising effect of freezing seems to be annulled when the meat is adequately aged prior to freezing. In the current study, our results show that the shear force (measurement of tenderness with peak force) of meat aged between 1 and 7 days did not differ (P > 0.05) across the diets. This is contrary to Grayson et al. (2014)’s report that tenderness increases with the degree to which the meat is aged prior to freezing. Although the meat WBSF show similarities amongst the diets regardless of aging period, numerically, the WBSF values of the 7-days aged meat were lower (average of 3.2 kg) compared to that of the 1-day aged meat (average of 3.9 kg). This indicates that aging increases meat tenderness. The similarities, moreover, indicate that inclusion of Napier grass in diets yielded tender meat according to the threshold level reported by Shackelford et al. (1999): ‘tough’ (> 4.6 kg) ‘intermediate’ (3.9–4.6 kg) and ‘tender’ (< 3.9 kg).

Table 9 demonstrates the sarcomere and myofibrillar fragmentation lengths of meat from steers on diets consisting of graded levels of Napier grass. The sarcomere and myofibrillar fragment lengths are used to indirectly evaluate the meat tenderness (Modika et al., 2019). Meat with shorter sarcomeres is tougher compared to that with longer sarcomeres (Haeger et al., 2020). According to Smili et al. (2022), the increase in myofibrillar fragmentation is an indication of the extent of tenderisation that has occurred. Hope-Jones and Strydom (2021) reported a relationship between MFL with sensory tenderness and shear force measurements. The authors stated that increased breaking down of myofibrillar proteins by collagenase leads to decreased shear force during ageing and shorter MFLs that are associated with a higher degree of proteolysis and meat tenderness (Basson et al., 2022). Additionally, MFL of more than 40 μm is correlated with tough meat (Hope-Jones and Strydom, 2021). In this study, the sarcomere and myofibrillar fragmentation lengths either aged for 1 or 7 days were similar (*P* > 0.05) across the dietary treatments (Table 9). This is an indication that proteolysis occurred in the same manner on meat from steers fed all the treatments diets, and thus resulted in tender meat. This suggests that dietary inclusion of Napier in steers fattening diets did not inhibit the proteolytic activity for breaking down meat fibres. The results indicate to Napier inclusion level not exceeding 300 g kg^− 1^ in steers fattening diets generating meat with long shelf life, tender and normal drip loss and colour.

### Meat proximate composition

Meat is a muscle tissue of a slaughtered animal composed of water, proteins, fat, minerals and a small quantity of carbohydrates (Shah et al., 2014). Water (moisture) is quantitatively the most vital component of meat consisting of approximately 75% of weight (Geletu et al., 2021). Protein is the most valued component in meat from the meat processing and nutritional point of view (Geletu et al., 2021). The meat fat portion comprises of some fat-soluble substances, such as vitamins (Ahmad et al., 2018). When heated, fat in meat acts as one of the precursors of flavour by combining with amino acids (building blocks of proteins) and other components (Suleimenova, 2016). The meat proximate composition (moisture, protein, lipid and minerals) and their ratio are important for the health of consumers (Jung et al., 2015). The proximate composition of meat in this study was higher (*P* < 0.05) in meat from steers fed diets with 0 inclusion of Napier grass than that with 600 g kg^− 1^ of the grass, but comparable to that with 300 g kg^− 1^ of the grass, except for the dry and organic matter contents (Table 10). Meat from steers fed diet containing 600 g kg^− 1^ Napier grass had high moisture content but low fat content, which is indirectly proportion.

## Conclusion

Napier grass can be incorporated in steers’ diet at not more than 300 g kg^− 1^ inclusion level. Inclusion level exceeding 300 g kg^− 1^ could reduce the growth performance of the steers, and compromise the quality of carcasses and meat. The most noticeable effect on increasing (i.e. > 300 g kg^− 1^) dietary inclusion of Napier grass in steers diet was the reduction of fat in the meat, which is something important for consumers.

## Data Availability

Not applicable.
